# Complex genetic background in a large family with Brugada syndrome

**DOI:** 10.14814/phy2.12256

**Published:** 2015-01-27

**Authors:** Siamak Saber, Mohamed‐Yassine Amarouch, Amir‐Farjam Fazelifar, Majid Haghjoo, Zahra Emkanjoo, Abolfath Alizadeh, Massoud Houshmand, Alexander V. Gavrilenko, Hugues Abriel, Elena V. Zaklyazminskaya

**Affiliations:** I. M. Sechenov First Moscow State Medical University, Moscow, Russia; Environment & Natural Substances Team, University of Sidi Mohamed Ben Abdellah‐Fes, Multidisciplinary Faculty of Taza, Taza, Morocco; Cardiac Electrophysiology Research Center, Rajaie Cardiovascular Medical and Research Center, Iran University of Medical Sciences, Tehran, Iran; Medical Genetics Department, National Institute for Genetic Engineering & Biotechnology, Tehran, Iran; Petrovsky Russian Research Center of Surgery, RAMS, Moscow, Russia; Department of Clinical Research, Ion Channels and Channelopathies, University of Bern, Bern, Switzerland

**Keywords:** Brugada syndrome, inherited channelopathy, *KCNH2*, Na_v_1.5, *SCN5A*

## Abstract

The Brugada syndrome (BrS) is an inherited arrhythmia characterized by ST‐segment elevation in V_1_–V_3_ leads and negative T wave on standard ECG. BrS patients are at risk of sudden cardiac death (SCD) due to ventricular tachyarrhythmia. At least 17 genes have been proposed to be linked to BrS, although recent findings suggested a polygenic background. Mutations in *SCN5A*, the gene coding for the cardiac sodium channel Na_v_1.5, have been found in 15–30% of index cases. Here, we present the results of clinical, genetic, and expression studies of a large Iranian family with BrS carrying a novel genetic variant (p.P1506S) in *SCN5A*. By performing whole‐cell patch‐clamp experiments using HEK293 cells expressing wild‐type (WT) or p.P1506S Na_v_1.5 channels, hyperpolarizing shift of the availability curve, depolarizing shift of the activation curve, and hastening of the fast inactivation process were observed. These mutant‐induced alterations lead to a loss of function of Na_v_1.5 and thus suggest that the p.P1506S variant is pathogenic. In addition, cascade familial screening found a family member with BrS who did not carry the p.P1506S mutation. Additional next generation sequencing analyses revealed the p.R25W mutation in *KCNH2* gene in *SCN5A*‐negative BrS patients. These findings illustrate the complex genetic background of BrS found in this family and the possible pathogenic role of a new *SCN5A* genetic variant.

## Abstract (precis)

We present the results of clinical, genetic, and expression studies of a large Iranian family with BrS carrying a novel genetic variant (p.P1506S) in *SCN5A*. These mutant‐induced alterations found in whole‐cell patch‐clamp experiments lead to a loss of function of Nav1.5 and thus suggest that the p.P1506S variant is pathogenic. Additional next generation sequencing analyses revealed the p.R25W mutation in *KCNH2* gene in *SCN5A*‐negative BrS family member.

## Introduction

The Brugada syndrome (BrS) is an inherited arrhythmia characterized by ST‐segment elevation in the right precordial leads on the ECG and is associated with an increased risk of sudden cardiac death (SCD) from polymorphic ventricular tachycardia and ventricular fibrillation (Brugada and Brugada 1992; Wilde et al. 2002; Antzelevitch et al. 2003). The prevalence of BrS is estimated between 1:2,000 and 1:100,000 in different countries (Antzelevitch et al. 2005; Probst et al. 2010; Risgaard et al. 2013). It is commonly accepted that BrS is inherited by an autosomal dominant mode with a strong male predominance (Antzelevitch et al. 2003).

Recently, seventeen susceptibility genes were identified for BrS. However, mutations in these genes account for only 35–40% of familial BrS (Nielsen et al. 2013), and 15–30% of the identified mutations are located in *SCN5A*, the gene encoding the cardiac sodium channel Na_v_1.5 (Probst et al. 2009; Kapplinger et al. 2010). Indeed, more than 370 mutations in the *SCN5A* gene were found in probands with BrS. Many of these were functionally characterized in cell lines expressing mutant Na_v_1.5 channel and have shown a loss‐of‐function effect on sodium current (The gene connection for the heart. http://triad.fsm.it/cardmoc/). However, it was found that some large BrS‐affected families contained *SCN5A*‐positive and *SCN5A*‐negative family members, and it put the question about direct causative role of *SCN5A* gene in BrS (Probst et al. 2009). Furthermore, recent findings suggest that common genetic variants in other genes might have a strong impact on the manifestation of genetic diseases such as BrS (Bezzina et al. 2013).

In the current study, we report a large Iranian family with BrS carrying an unusual combination of two rare genetic variants, p.P1506S in the *SCN5A* gene and p.R25W in the *KCNH2* gene.

## Material and Methods

The present study was performed in accordance with the Helsinki Declaration and local ethics committee. Written informed consent for clinical and genetic evaluation was obtained from each member of the family. Some family members have declined detailed clinical examinations or genetic testing after the first consultation, and their clinical and genetic data are presented when available.

### Clinical evaluation

The clinical follow‐up included a review of personal and familial history complete physical examination, 12‐lead ECG, 24‐hour Holter ECG monitoring, and echocardiography. A flecainide (400 mg/oral) challenge test was performed for three family members (II.3, III.1, and III.9) as described in (Benito et al. 2009).

### Genetic study

Genetic screening for mutations in *SCN5A* gene was performed by PCR‐based bidirectional capillary Sanger sequencing. Originally designed oligonucleotide primers encompassed all 28 exons and ~150‐bp adjacent intronic areas. The primers can be provided on request. Cascade familial screening for genetic variants of interest was performed by PCR‐RFLP. The prevalence of new genetic variants was assessed in a group of 100 healthy ethnically matched volunteers (200 chromosomes). An additional genetic study was performed by semiconductor sequencing platform Personal Genome Machine (PGM Ion Torrent; Thermo Fisher Scientific Brand, Waltham, MA) based on multiplex PCR primer pools designed by Ampliseq designer service. Mutation screening was performed for *KCNQ1, KCNE2, KCNE3, KCNE1, KCNJ2, KCNH2, SCN5A, SCN1B, SCN3B, SCN4B,* and *SNTA1* genes.

### Site‐directed mutagenesis

Site‐directed mutagenesis was performed on pCDN3.1‐hSCN5A using the Quick‐Change II XL site‐directed mutagenesis kit (Stratagene, La Jolla, CA) according to the manufacturer's instructions. The resulting Na_v_1.5 protein is a splice variant lacking a glutamine at position 1077 (Q1077del). Thus, the P1506S substitution corresponds to the P1505S in the used *SCN5A* sequence.

### Cellular electrophysiology

Human Embryonic Kidney 293 cells (HEK293) were cultured at 37°C in Dulbecco's Modified Eagle's Medium (DMEM) supplemented with 10% FBS (fetal bovine serum), 4 mmol/L glutamine, and a cocktail of streptomycin–penicillin antibiotics in a humidified atmosphere of 5% CO_2_ and 95% air. All cell medium components except glutamine (Sigma–Aldrich) were purchased from Gibco. The HEK293 cells were transfected with DNA complexed to JetPEI (Polyplus‐transfection) according to the manufacturer's instructions. DNA concentrations were 1 *μ*g of pCDN3.1‐ Na_v_1.5 wild type (WT), p.P1505S and 1 *μ*g of pIRES‐h*β*1‐CD8. Six to eight hours after transfection, the cells were isolated and seeded in plastic petri dishes at low density. Twenty‐four hours after transfection, the resulting sodium current (*I*_Na_) was recorded at room temperature (23–25°C) under whole‐cell voltage‐clamp conditions with an Axopatch 200B (Axon Instruments, Inc., Jakarta Selatan, Indonesia) amplifier interfaced to a personal computer driven by the PClamp 10 software (Molecular Devices Corporation, Sunnyvale, CA). The leak and capacitive currents were canceled using a P/4 protocol. The cells were bathed with an extracellular solution containing (in mmol/L): NaCl 50, NMDG‐Cl 80, CsCl 5, MgCl_2_ 1.2, CaCl_2_ 2, HEPES 10, glucose 5. The pH was adjusted to 7.4 with CsOH. Glass pipettes (tip resistance: 1.3–2.5 MΩ) were filled with an intracellular medium containing (in mmol/L): CsCl 60, aspartic acid 50, CaCl_2_ 1, MgCl_2_ 1, HEPES 10, EGTA 11, Na_2_ATP 5. pH was adjusted to 7.2 with CsOH. All products were purchased from Sigma.

### Data analysis and statistical methods

Currents were analyzed with Clampfit software (Axon Instruments, Inc). Data were analyzed using a combination of pClamp10, Excel (Microsoft, Redmond, WA) and Prism (Graphpad, San Diego, CA).

Comparisons between groups were performed with two‐tailed Student's *t* test or two‐way ANOVA for normally distributed parameters. Data are expressed as mean ± SD. A *P*‐value <0.05 was considered statistically significant.

## Results

**Figure 1.**
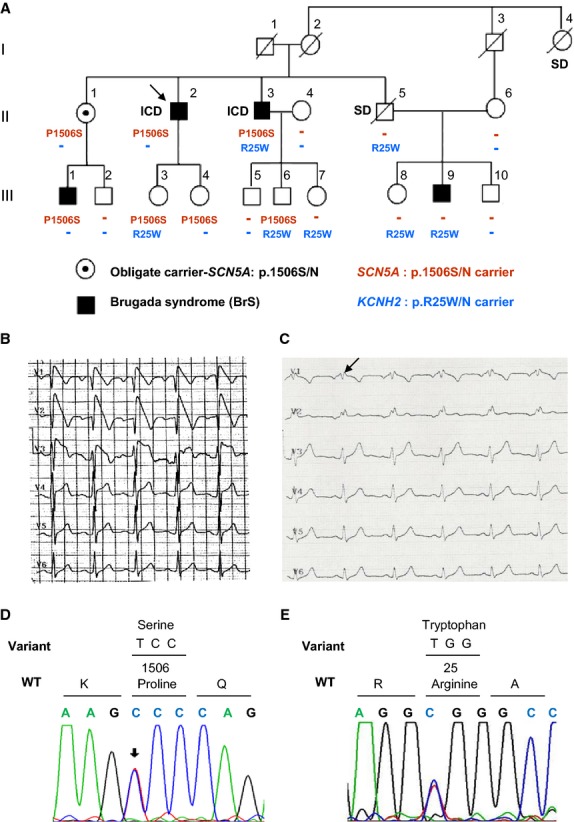
(A) The pedigree, phenotypes, and mutation status of family members. Males and females are indicated by squares and circles, respectively. The arrow shows the proband. SD: sudden death. ICD: implantable cardioverter defibrillator. (B) Representative 6‐lead (V1–V6) surface ECG of the proband showing a typical Brugada syndrome (BrS) pattern. (C) Representative 6‐lead surface ECG of the III.9 individual with a BrS type 2 pattern. (D) Chromatogram fragment of exon 26 of *SCN5A* gene. c.4516C>T heterozygote condition was shown by arrow and change the Proline to Serine in codon 1506 of *SCN5A* gene. (E) Chromatogram fragment of *KCNH2* gene. c.73C>T heterozygote condition was shown by arrow and change the Arginine to Tryptophan in codon 25 of *KCNH2* gene.

This study included 15 members of a large Iranian family with BrS and a history of SCD (the pedigree is shown on Fig. 1A, Tables 1 and 2).

**Table 1.** Susceptibility genes for Brugada syndrome

	Gene	Type of BrS	Detection rate of mutation (%)
The cardiac voltage‐gated sodium channel and associated proteins	*SCN5A*	BrS 1	11–28
	*GPD1‐L*	BrS 2	<1
	*SCN1B*	BrS 5	<1
	*SCN3B*	BrS 7	<1
	*MOG1*	BrS 11	<1
	*SLMAP*	BrS 15	<1
	*SCN2B*	BrS 17	<1
Potassium channels and associated proteins	*KCNH2*	BrS 8	<1
	*KCNE3*	BrS 6	<1
	*KCNE5*	BrS 12	<1
	*KCNJ8*	BrS 9	<1
	*KCND3*	BrS 13	<1
Calcium channel and associated proteins	*CACNA1c*	BrS 3	3–4
	*CACNB2b*	BrS 4	2–3
	*CACNA2D1*	BrS 10	<1
Nonselective cation channels	*TRMP4*	BrS 16	<6
Hyperpolarization activated cyclic nucleotide‐gated channels	*HCN4*	BrS 14	<1

BrS, Brugada syndrome.

**Table 2.** Clinical and genetic data of the family members

	Sex	*SCN5A* p.P1506S	*KCNH2* p.R25W	Brugada pattern on 1st ECG testing		Syncope	HR	PR msec	QTc msec	ICD
				Spontaneous	After PCT					
II.2 Proband	M	+/−	−/−	BrS 1	NP	+	93	180–200	425	+
II.3	M	+/−	+/−	−	+	+	78	180–200	404	+
II.5	M	−/−	+/−	−	Negative	+	NA	NA	NA	No
II.6	F	−/−	−/−	−	NP	−	67	160–180	428	No
III.1	M	+/−	−/−	BrS 1/BrS 2	+	−	78	200–220	413	R
III.2	M	−/−	−/−	−	NP	−	89	160	395	No
III.3	F	+/−	+/−	−	Refused	−	NA	180–200	390	No
III.4	F	+/−	−/−	−	Refused	−	NA	NA	NA	No
III.5	M	−/−	−/−	−	R	−	97	160–180	439	No
III.6	M	+/−	+/−	−	R	−	65	160–180	428	No
III.7	F	−/−	+/−	−	R	−	95	160–180	363	No
III.8	F	−/−	+/−	−	R	−	73	140–160	404	No
III.9	M	−/−	+/−	−	+	+	81	140–160	380	R
III.10	M	−/−	−/−	−	−	−	100	180–200	405	No

BrS, Brugada syndrome; +/−, present in heterozygous state; −/−, genetic variant is absent; NA, not available; NP, not performed; R, recommended; Refused, test was recommended but refused.

The proband, a 48‐year‐old male, had been admitted to a hospital because of recurrent syncopes related to night eating and permanent dizziness. The first syncope had occurred at 23 years of age. Two close relatives died suddenly at the ages of 36 (I.4) and 54 (II.5).

Brugada syndrome diagnosis was considered based on spontaneous Brugada pattern on baseline ECG, with ST‐elevation in V_1_–V_3_ followed by negative T wave, and maximal J‐point elevation >7 mm in V_2_ (Fig. 1B). No structural abnormality, valvular disease, or decreased contractility was found by echocardiography. An ICD was implanted in 2012 and one appropriate shock was registered during the 1‐year follow‐up. No anti‐arrhythmic medication was administered. Genetic analysis of *SCN5A* gene revealed a novel missense variant c.4516C>T leading to the p.P1506S substitution (Fig. 1D). This variant was not found in a control group of 100 ethnically matched healthy volunteers (200 alleles). Clinical evaluations and cascade genetic screening for the p.P1506S variant were performed for an additional 13 relatives. BrS was suspected in five family members (accordingly to a first ECG). For three of them (II.3, III.1, and III.9), BrS diagnosis was made on the basis of a positive flecainide challenge test. Two patients with suspected diagnosis of BrS (III.3 and III.4) declined pharmacological testing. An ICD implantation was performed for the patient II.3. This patient did not receive any anti‐arrhythmic drugs and no appropriate shock has been recorded during 1 year after implantation.

Mutation screening in exon 26 of *SCN5A* revealed the missense variant p.P1506S in five family members (II.3, III.1, III.3, III.4, and III.6). Surprisingly, we did not find the p.P1506S variant in one relative (III.9) with a clear BrS pattern and presyncopal episodes in anamnesis (Fig. 1C); his father (II.5) died suddenly at 54 years of age during a third syncope.

Additional genetic screenings were performed for *KCNQ1, KCNE2, KCNE3, KCNE1, KCNJ2, KCNH2, SCN5A, SCN1B, SCN3B, SCN4B,* and *SNTA1* genes for *SCN5A*‐negative BrS patient. The genetic variant c.73C>T in *KCNH2* gene isoform C (hERG‐b), leading to the p.R25W substitution, was found (Fig. 1F). Repeated cascade family screening for this variant was carried out for other family members, and it was detected in 7 of them (II.3, II.5, III.3, III.6, III.7, III.8, and III.9). Clinical and genetic findings from the family members are summarized in the Table 1.

**Figure 2.**
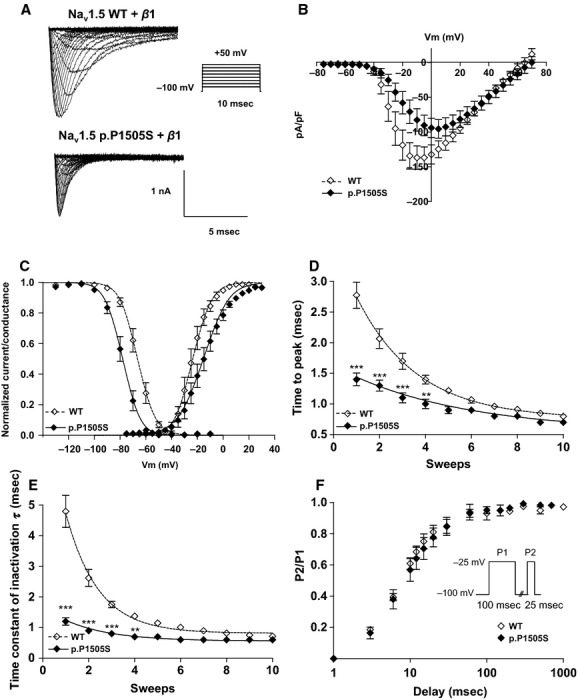
(A) Current traces obtained with inset protocol from Na_v_1.5‐WT and Na_v_1.5‐P1505S transfected HEK293 cells. (B) Current‐voltage traces of the Na_v_1.5‐WT and Na_v_1.5‐P1505S channels. (C) Steady‐state activation and inactivation curves. Activation properties were determined from I/V relationships by normalizing peak I_N__a_ to driving force and maximal *I*_Na_. Parameters for the voltage‐dependence steady state of activation and steady state of inactivation (20‐msec test pulse to ‐10 mV after a 500 msec conditioning prepulse). (D) Sodium current time‐to‐peak values were used to evaluate the activation kinetics. (E) Fast inactivation time constants were measured by fitting the inactivation phase of the Na^+^ current to a single exponential equation for WT and p.I141V. For D and E, to avoid being biased by the positive shift of the voltage dependence of activation, for each individual cell, the first analyzed potential (sweep 1) was corresponding to the potential for which the amount of the activated channels is more than 20%. For the statistical analysis, a two‐way ANOVA test followed by a Bonferroni correction was used to compare point by point the measured activation and inactivation ***, *P* < 0.001, **<0.01 versus WT. (F) Recovery from fast inactivation was measured using a twin‐pulse protocol (in inset).

To investigate the functional consequences of the p.P1505S mutation on Na^+^ channel activity, we used the whole‐cell configuration of the patch‐clamp technique. The presence of the mutation reduced the peak current density of Na_v_1.5 (current density at ‐20 mV (pA/pF): −119 ± 21, *n* = 6, versus −58 ± 16, *n* = 8, **P < 0.01; for WT and mutant channels, respectively, Fig. 2A and B). The voltage dependence of steady state of activation was shifted toward more positive potentials (V_1/2_ act: −24 ± 2 mV, *n* = 6, vs. −15 ± 2.8 mV, *n* = 8, *P < 0.05; slope: 6 ± 0.3 mV vs. 8.8 ± 0.6 mV, **P < 0.01; for WT and mutant channels, respectively, Fig. 2C). In addition, the voltage dependence of steady state inactivation was shifted toward more negative potentials in the presence of the p.P1505S mutation (V_1/2_ act: −66 ± 2 mV, *n* = 6, vs. −80 ± 4 mV, *n* = 8, **P < 0.01; slope: 6.1 ± 0.5 mV vs. 5.6 ± 0.2 mV; for WT and mutant channels, respectively, Fig. 2C). For a given command voltage, the activation and inactivation kinetics were hastened in the mutant channel (Fig. 2D and E) However, no significant differences were observed regarding the recovery from fast inactivation (t1/2 of recovery from inactivation: 9.4 ± 1.3 msec, *n* = 4, versus 9.3 ± 1 msec, *n* = 7; Fig. 2F).

## Discussion

This study reports on a large Iranian family with BrS where the proband and several of its relatives carry a novel missense mutation p.P1506S in the *SCN5A* gene. PQ interval prolongation >200 msec in patients with Brugada syndrome caused by *SCN5A* mutations has been described previously (Smits et al. 2002). The PQ interval duration in *SCN5A‐*positive family members was significantly longer than in noncarriers (Table 3), and we consider this fact as an indirect evidence of the association of the p.P1506S variant with a clinical phenotype. In the presence of this mutation, the electrophysiological study revealed a significant loss of function of sodium current related to a destabilization of the open conformation of the Na_v_1.5 channel. This effect is reflected by a positive shift of steady state of activation, a negative shift of steady state of inactivation, and an acceleration of the inactivation kinetics. Reduction in sodium current is typical for known *SCN5A* mutations leading to BrS, and we suggest that the new missense mutation p.P1506S is linked with the described BrS in this family.

**Table 3.** Evaluation of PR interval durations in *SCN5A*‐positive versus *SCN5A*‐negative family members

Genetic variant	Number of carriers	PR msec	*P*‐value
*SCN5A* p.P1506S/N	6	190 ± 14.1	0.01
*SCN5A N/N*	4	156 ± 11.5	

However, one affected family member did not carry this mutation and this discrepancy in segregation needs additional explanation. Similar clinical observations of three independent families with genotype‐negative phenotype‐positive patients was previously reported by Probst et al. (2009) suggesting that, in some cases, loss‐of‐function *SCN5A*‐mutation might not be sufficient to cause BrS but could act as a predisposing or a modulating factor. This suggestion was supported by the recent study by Bezzina et al. (2013) showing that common genetic variants can have a strong impact on the manifestation of BrS.

The coexistence of two independent mutations in the same family is common in cardiac channelopathies patients. The prevalence of compound heterozygosity has been reported to amount from 4% to 7.9% in several cohorts of LQTS patients, and the carriage of three independent mutations was found in about 1% of genotype‐positive patients (Zaklyazminskaya and Abriel 2012).

The spectrum of genes causing LQTS and BrS is similar and a similar prevalence of two independent mutations in BrS cohorts may also be expected. In the current pedigree, we propose that *SCN5A*‐negative affected family member may have BrS caused by mutations in another gene.

We have used semiconductor sequencing PGM Ion Torrent platform to resequence the *SCN5A* gene and sequenced 10 additional susceptibility genes for BrS. A missense mutation p.R25W in *KCNH2* has been found in *SCN5A*‐negative BrS patient.

The *KCNH2* gene encoded the alpha‐subunit of the rapid delayed rectifier K^+^ channel (*I*_Kr_). Most of known mutations in *KCNH2* gene were associated with LQTS and SQTS, and only a few variants were found in BrS patients (Verkerk et al. 2005; Itoh et al. 2009; Nielsen et al. 2013; Wang et al. 2014). The hERG1b‐p.R25W variant has been shown to induce a loss of function of hERG channel and was associated with intrauterine unexplained fetal death (Crotti et al. 2013). Nevertheless, taking into account the hypothesis that p.R25W presented in *hERG‐b* isoform might affect the QT interval duration, we compared the corrected QT interval values in *KCNH2*‐positive and *KCNH2*‐negative family members (Table 3). The duration of QTc in patients carrying only the *KCNH2*‐p.R25W variant was significantly shorter than in those who carried only the *SCN5A* mutation (*P* < 0.05) (Table 4). Recently, it was shown that QTc duration in *KCNH2*‐related patients tends to be shorter comparing with QTc in *SCN5A*‐related Japanese BrS patients (Ohno et al. 2011).

**Table 4.** Evaluation of QTc interval durations in patients with combinations of *KCNH2* and *SCN5A* variants

Genetic variants combination	Number of carriers	QTc msec
*SCN5A* p.P1506S/N and *KCNH2 N/N*	3	419 ± 8.4
*SCN5A* p.P1506S/N and *KCNH2* R25W/N	3	407 ± 19.2
*SCN5A N/N* and *KCNH2* p.R25W/N	4	382 ± 20.5

There was no significant difference in QTc duration between patients with one genetic variant versus double mutation carriers in our study.

It may be proposed that the p.R25W variant in the *KCNH2* gene might be a causative variant in the *SCN5A*‐negative BrS family member.

## Conclusion

Here, we presented a large family where two independent rare genetic variants were identified in patients with BrS. A new missense mutation p.P1506S in the *SCN5A* gene leads to a significant reduction in Na^+^ current related to a destabilization of the open conformation of Na_v_1.5. We propose that the p.P1506S mutation in *SCN5A* is causative for BrS in most of the affected family members. Rare genetic variant p.R25W in *KCNH2* gene might be responsible for BrS phenotype in *SCN5A*‐negative affected family members. The precise molecular mechanism leading to BrS for this variant remains to be elucidated. Further genetic and functional studies analyzing genotype–phenotype correlations in BrS are required. We illustrate that the concept by default “one family‐one phenotype‐one causative genetic variant” is not always applicable for families with cardiac channelopathies, and comprehensive genetic testing can be important for family screening and genetic counseling.

## Conflicts of Interest

None declared.
